# Computed Tomography Osteodensitometry for Assessment of Bone Mineral Density of the Canine Head—Preliminary Results

**DOI:** 10.3390/ani11051413

**Published:** 2021-05-14

**Authors:** Glynn Woods, Nicolas Israeliantz Gunz, Ian Handel, Tiziana Liuti, Richard J. Mellanby, Tobias Schwarz

**Affiliations:** Royal (Dick) School of Veterinary Studies and Roslin Institute, The University of Edinburgh, Roslin EH25 9RG, UK; N.Israeliantz-Gunz@sms.ed.ac.uk (N.I.G.); ian.handel@ed.ac.uk (I.H.); Tiziana.Liuti@ed.ac.uk (T.L.); Richard.Mellanby@ed.ac.uk (R.J.M.); Tobias.Schwarz@ed.ac.uk (T.S.)

**Keywords:** bone marrow density, osteodensitometry, bone marrow, computed tomography

## Abstract

**Simple Summary:**

Metabolic bone disease can have disastrous consequences on canine health. Unlike in human medicine where awareness of osteoporosis and bone mineral density (BMD) disorders have led to the holistic application of osteodensitometry, application of osteodensitometry in dogs is limited. We aimed to assess the utility of quantitative computed tomography (qCT) bone mineral density (BMD) measurement of the canine calvarium using semiautomated osteodensitometry software and define host factors associated with canine BMD in a skeletally healthy population. Calvarium qCT can be used to rapidly obtain BMD measurement of dogs. Canine BMD was negatively associated with weight, whereas there was no relationship between BMD and age or sex. Many chronic canine diseases can significantly affect bone health via a variety of pathophysiological mechanisms. This efficient qCT method could facilitate rapid BMD screening in dogs undergoing CT evaluation and ultimately encourage further BMD investigation.

**Abstract:**

Despite bone mineral density (BMD) being regularly measured in human patients, BMD studies in clinical cohorts of dogs is lacking. In order to facilitate BMD assessment and in turn better identify dogs suffering from metabolic bone disease, rapid, easy and precise computed tomography (qCT) techniques are required. In this study we aimed to assess the utility of quantitative computed tomography (qCT) bone mineral density (BMD) measurement of the canine calvarium using a semiautomated osteodensitometry software and define host factors associated with canine bone mineral density in a skeletally healthy population. Calvarial qCT at the level of the temporomandibular joints was performed on 323 dogs using a dedicated osteodensitometry calibration phantom during a clinically indicated head computed tomography (CT). Calvarial BMD was analyzed using a dedicated semiautomatic osteodensitometry software for contouring of the calvarial lamellar bone margins and BMD calculation. The mean duration of the calvarial qCT scanning was 64.6 s, and the mean duration of BMD analysis was 34 s, with a mean of two manual adjustments required for the bone margin tracing. The median BMD of all dogs in our study was 659 mg Calcium hydroxyapatite/mL. There was a negative linear correlation between BMD and body weight, but no correlation with age, sex or neutered status. Canine BMD assessment using qCT of the calvarium is a practical and fast technique that can be added to a clinical CT examination with minimal extra time requirements. Canine BMD host-dependent factors exhibit different relationships from that of humans; however, further investigation is warranted.

## 1. Introduction

Metabolic bone disease (MBD) is an important cause of morbidity in dogs. Numerous metabolic diseases can cause skeletal pathology including primary hyperparathyroidism and secondary hyperparathyroidism resulting from nutritional deficiencies or renal disease [1,2,3]. In extreme cases, MBD can cause bony hyperaesthesia, bone loss (osteopenia) and pathologic fractures [4,5]. 

Despite the devastating impact MBD can have on the welfare of dogs, it remains challenging to accurately and rapidly assess skeletal health in most veterinary care settings. This contrasts with the human sector where the assessment of skeletal health by the evaluation of bone density using dual energy x-ray absorptiometry (DEXA) scans or computed tomography (CT)-based technologies are well established techniques [6]. These techniques are regularly incorporated into health metrics that can help guide medical and nutritional interventions for a variety of skeletal and extra-skeletal conditions [7,8,9]. Both modalities are heavily employed in the screening of osteoporosis [10]. Use of these techniques provides clinicians with an opportunity to detect bone mineral density changes early in a disease process and initiate therapy to reduce future morbidity [11].

Historically, radiographs were the modality of choice to evaluate bone density and diagnose osteopenia in dogs. However, the sensitivity of radiography to detect bone density abnormalities is poor, with at least 40% of bone loss occurring prior to radiographic detection [12]. Radiographic interpretation is also subjective and influenced by technical and operator factors [13]. Only a small number of studies have assessed bone density using DEXA and qCT protocols in animals [14,15,16]. To date, studies investigating bone density in dogs have typically utilized human methodologies, focused on small research populations using techniques originally designed for people in which the femur and lumbar vertebrae are used to assess bone density [14,17]. However, bone mineral density (BMD) changes precipitated by extra-skeletal disease are not restricted to lumbar vertebrae [1,2,18]. In fact, bones of the skull are particularly susceptible to effects of nutritional deficiencies in both humans and dogs and contribute toward pathological fractures and cause “rubber jaw,” which results from the replacement of mandibular bone with fibrocartilaginous tissue [19,20,21,22,23]. A recent report of nutritional secondary hyperparathyroidism in a dog highlighted that skull bone density can be lower in dogs fed a nutritionally incomplete diet [2]. The possibility of employing CT to assess BMD of the skull bones in dogs is an appealing concept. It would circumvent the need for laborious positioning, reduce anesthetic time or avoid general anesthesia and has the potential to become the standard of practice for dogs undergoing CT for clinical diagnostics.

We hypothesized that osteodensitometric qCT of the canine head will be fast to perform in sedated or anaesthetized dogs; that BMD results can be fast and easily calculated; and that lower weight, higher age, female dogs and neutered dogs will be negatively associated with BMD.

Here we report a novel approach to qCT BMD measurement in the calvarium of a cohort of dogs without skeletal disease. We have then used this technique to define the host factors associated with canine BMD in dogs with no clinical signs of skeletal disorders.

## 2. Materials and Methods

### 2.1. Animal Selection 

This study was a prospective clinical cohort study performed on dogs undergoing CT examination for a primary clinical reason for a non-skeletal disease at the Royal (Dick) School of Veterinary Studies’ Hospital for Small Animals. Medical and imaging records from dogs that underwent a general anesthesia or sedated computed tomography (CT) examination that included a qCT examination of the head between May 2015 and July 2019 were prospectively gathered. The study was ethically approved by the University of Edinburgh’s Veterinary Ethical Research Committee (institutional reference number VERC 99 14). 

Computed tomography studies were all initially clinically evaluated by a senior board-certified veterinary radiologist (T.S. and T.L.). Studies with non-diagnostic quality in which BMD could not be calculated were excluded first and causes of exclusion were recorded. Dogs with evidence of intranasal disease extending to the skull and intracranial disease involving the hypothalamus pituitary axis structures were also excluded from BMD assessment. Additional exclusion criteria for the study included any medical history or suspected underlying pathology that could predispose the dog to generalized bone disease, metabolic disorders or malignancy, trauma or implants at the measurement sites, or any medical treatments with progestogens, steroids or bisphosphonates that are known to affect bone metabolism and density. Dogs below the age of 12 months were excluded.

### 2.2. Quantitative CT Examination

**Figure 1:**
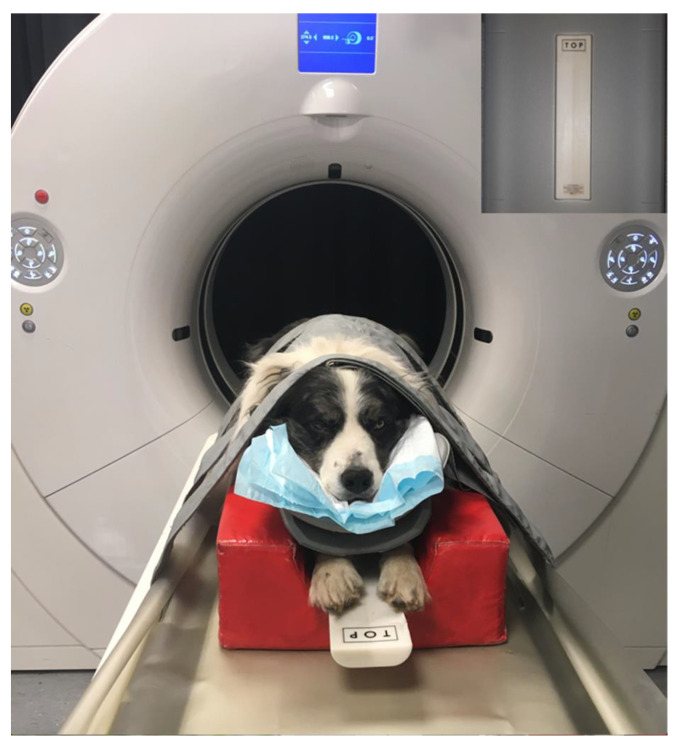
Photo of a sedated dog in ventral recumbency on the CT table with the osteodensitometry phantom placed beneath. The phantom is easy and quick to position either on the table as seen here or in a dedicated slot within the cushioning material of the CT patient table (see inset upper right corner).

Computed tomography images of the head were acquired by use of a 4-slice helical CT scanner (Volume Zoom^®^, Siemens, Erlangen, Germany) before October 2016 and a 64-slice helical CT scanner (Somatom^®^ Definition AS Siemens, Erlangen, Germany) after that time with a dedicated osteodensitometry calibration phantom (model number 8783219, Siemens, Munich, Germany). A helical CT scan without contrast medium application was performed as part of all dogs’ diagnostic investigation under sedation or general anesthesia. Dogs were kept in sternal recumbency throughout the scan. The dog was not moved after scanning was initiated. The examinations were performed by various imaging diagnostic residents assisted by an experienced radiographer and supervised by two authors of the study (T.S or T.L.). The osteodensitometry phantom was placed on the table under the head of all dogs (Figure 1). The osteodensitometry phantom contains one chamber with demineralized water (reference value 0 Hounsfield units (HU) and one chamber with calcium hydroxyapatite (CaHAP) with a concentration of 200 mg/mL. 

The CT table height was adjusted to the gantry center (125 mm table height) according to the manufacturer’s guidelines to ensure repeatability for all forthcoming BMD measurements. The table location of each dog’s temporomandibular joint was determined by one author (T.S.) and one additional single slice CT scan with 10 mm slice thickness was obtained at the level of the temporomandibular joint with the following settings: 80 kV, 1 s tube rotation time, 10 mm slice collimation, 512 × 512 matrix, 250 mm image reconstruction diameter. The electric current was automatically and individually selected by an automatic exposure control system (Care Dose 4D, Siemens Medical Solutions, International, Erlangen, Germany) depending on the body size and shape on the topogram scan. This resulted in different mAs settings between the dogs (range 20 to 150 mAs). The image was then reconstructed with 10 mm slice width using a dedicated reconstruction kernel (S80s, Siemens, Erlangen, Germany). The osteodensitometry function was identical between both CT units, and hence the technical factors for the CT scans were the same. All images acquired were stored on a local archiving and communication software. All studies were reviewed on an Apple Mac Pro^®^ (Apple, Cupertino, CA, USA) with a calibrated flat screen monitor, using a dedicated, open-source DICOM viewer software (Horos, Purview, Annapolis, MD, USA, version 3.3.6). 

**Figure 2:**
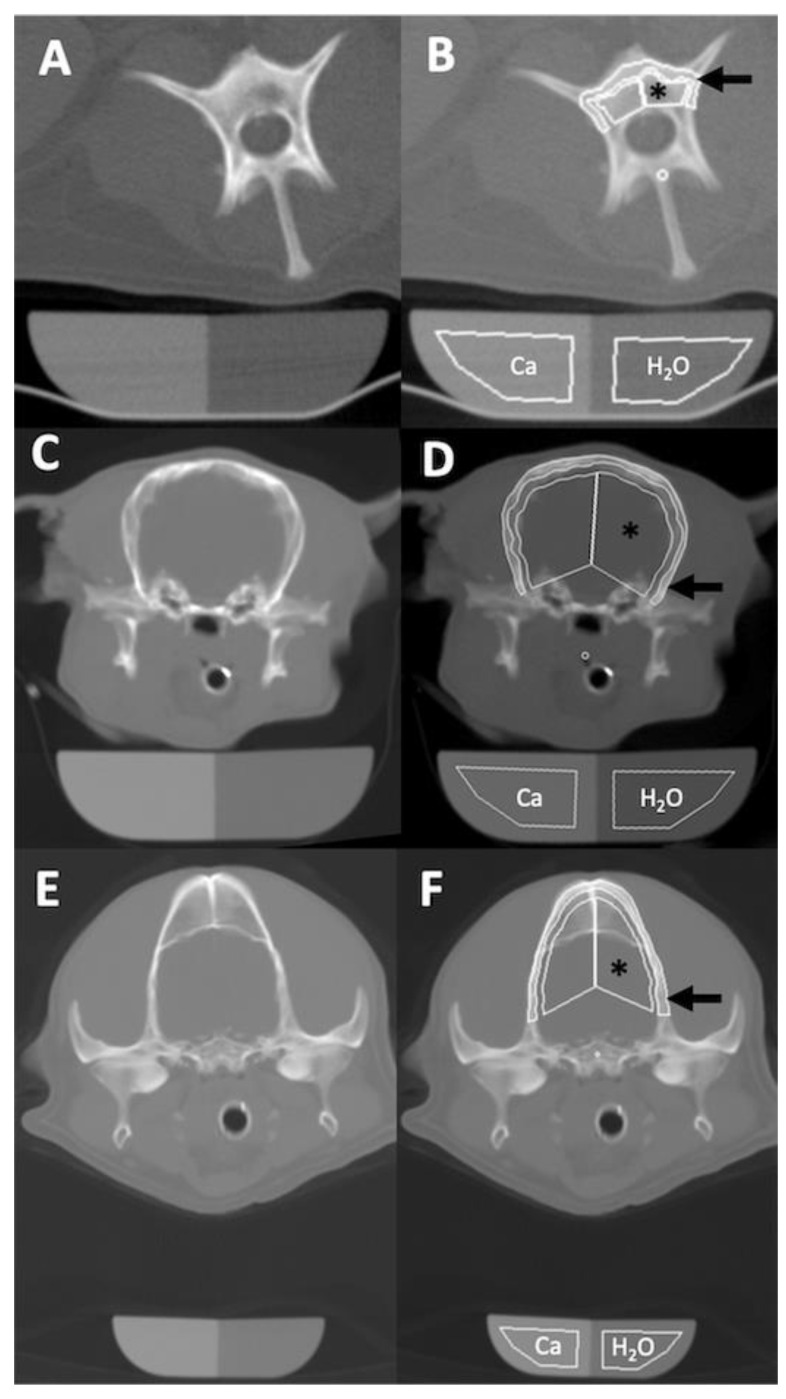
(**A**) Osteodensitometry CT image with 10mm slice width of the third lumbar vertebra of a mixed breed dog in dorsal recumbency and the osteodensitometry phantom beneath it. (**B**) Osteodensitometry bone margin contouring software (Osteo, Siemens, Erlangen, Germany) applied as designed, contouring the peripheral cortical (arrow) and central trabecular bone (*) and placing regions of interest over the water (H_2_O) and Calcium hydroxyapatite (Ca) chambers of the phantom. The calculated bone mineral density was 581.9 mg CaHAP/mL for the cortical and 359 mg CaHAP/mL for the trabecular bone. (**C**) Osteodensitometry CT image of the head of a Japanese Chin dog at level of the temporomandibular joints in ventral recumbency and with (**D**) osteodensitometry software applied. The peripherally contoured rim (arrow) represents the calvarial lamellar bone with a calculated bone mineral density of 503.4 mg CaHAP/mL. The results of the centrally contoured area (*) were discarded. (**E**) Osteodensitometry CT image of a Cocker Spaniel with (**F**) a calculated lamellar bone mineral density of 648.6 mg CaHAP/mL. The central measurement area contains diploë and brain tissue and was disregarded.

BMD was analyzed using a dedicated semiautomatic contouring software (Osteo, Siemens, Erlangen, Germany) that is designed to identify the cortical and trabecular bone of a lumbar vertebra (Figure 2A,B) and calculates mean BMD in mg/mL according to the following formula:

(1) $$\mathrm{Mean}\;\mathrm{BMD}=200\;\mathrm{mg}/\mathrm{mL}\;\frac{\mathrm{HUt}}{\mathrm{HUb}-\mathrm{HUw}}$$

where HUt is the measured density of the bone in HU, HUb is the measured density of the CaHAP solution of the phantom in HU, HUw is the measured density of demineralized water of the phantom in HU and 200 mg/mL is the concentration of the CaHAP solution in the phantom. The software-generated cortical bone traces were applied to lamellar bone of the calvarium, manually adjusted and measured the squamous part of the temporal and parietal bone at the level of the temporomandibular joint. The software-generated trabecular bone traces were attempted to be adjusted to the diploë (trabeculated skull bone) of the calvarium (Figure 2C–F). The BMD analysis was performed by qualified radiographers and radiologists who were specifically trained on the use of this software. Calculated mean BMD values were recorded for each dog.

In order to assess the ease and speed of the CT osteodensitometry scanning procedure and the bone mineral density calculation, 15 CT studies were randomly selected. In these studies, the time of acquisition of the last image of the last CT series prior to the qCT study and the acquisition time of the last qCT image were recorded and the time difference was calculated. The duration of the measurement process and the number of corrections applied using the osteodensitometry contouring software were also recorded. 

### 2.3. Statistical Analysis

Statistical analyses were performed using the R Statistical System [24]. Analysis of the association between mean and sd bone density-dependent variables and age, body weight, sex and neuter status predictors was performed using multivariable linear regression and including CT machine as a covariate. A subset of 40 dogs of similar weight and breed were selected to compare the effect of CT machine on osteodensitometry measurement. Re-estimation of the regression model including CT unit was performed and it was concluded that the CT unit was not a significant predictor and that estimates of significant effects did not change. Furthermore, direct comparison between each CT unit’s osteodensitometry measurement was performed using a subset of 40 similar breed dogs of similar weight and demonstrated overlap of confidence intervals.

For analysis of sex-related differences, the dogs were coded into four groups: intact males, intact females, neutered males and neutered females. Initially models were fitted using age, weight and sex variables. Then variables with effects that were not significantly different from zero were removed. Then, the effect on significant variables of adding back in removed variables was assessed. T-tests were used to determine significance of individual predictors, with *p*-values < 0.05 considered significant. As continuous predictors (age and weight) may have a non-linear relationship with mean BMD, linear models were then compared using models in which significant continuous predictors were divided into categories by quartile. Final model residuals were assessed for normality using QQ plots and models with residuals deviating from normality were re-estimated without the relevant observations to assess the impact. For osteodensitometry CT scanning and BMD measurement duration, the mean time span with 95% confidence interval was calculated. For BMD measurements, the mean number of corrections with 95% confidence interval was calculated. 

## 3. Results

### 3.1. Population Data

The CT osteodensitometry examinations of 448 dogs were initially gathered. A total of 48 dogs (10.7%) were excluded due to insufficient image quality of the qCT image to perform the BMD calculation. This was due to absence of the osteodensitometry phantom in the qCT image (16 dogs) or inappropriate positioning (32 dogs). Inappropriate positioning included slice location out with the level of the temporomandibular joints, excessive obliquity of the head or the phantom, and an excessive distance between the head and the phantom. A further 77 dogs were excluded due to age at time of qCT (<12 months); pathology of the head at the level of the BMD qCT slice; the presence of history, clinical exam and biochemical findings consistent with a pathology reported to affect BMD; detection of metastatic neoplastic disease and the recent use of glucocorticoid or bisphosphonate treatment. Two were excluded from the regression modelling as they had missing weight data. Hence a total of 323 dogs were included in the study. One hundred and ninety-two dogs were male, of which 120/192 (62.5%) were neutered. One hundred and thirty-one dogs were female, of which 98/131 (74.8%) were neutered. There was a large variation in the breeds of dog undergoing CT. The 5 most common breeds in this referral cohort were Labrador Retriever (26), Bulldog (22), Cocker Spaniel (16), Springer Spaniel (15) and cross breed (10). The median age of all dogs was 7 years (range 1–13.9 years). The median weight of the dogs included was 20 kg (range 1.7–59.8 kg). 

### 3.2. Osteodensitometry Procedure

It was possible to obtain a diagnostic-quality lamellar calvarial bone osteodensito-metry in all 323 dogs included in the study. Diploë was only present in a small number of dogs, and could not be measured accurately due to averaging with brain tissue and this was therefore not recorded (Figure 2E,F). The CT osteodensitometry scanning procedure had a mean duration of 64.6 s (95% confidence interval of 44–85 s). The CT osteodensitometry BMD measurement procedure was obtained with a mean number of 2 corrections (95% confidence interval of 1.4 to 2.0 corrections) and had a mean duration of 34 s (95% confidence interval of 28.9–39.1 s).

### 3.3. Bone Mineral Density Analysis

**Figure 3:**
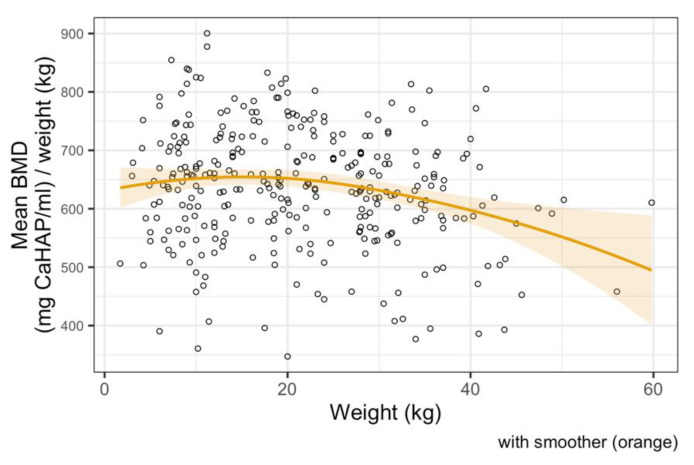
Linear regression model between bone mineral density and body weight, showing a negative correlation.

**Figure 4:**
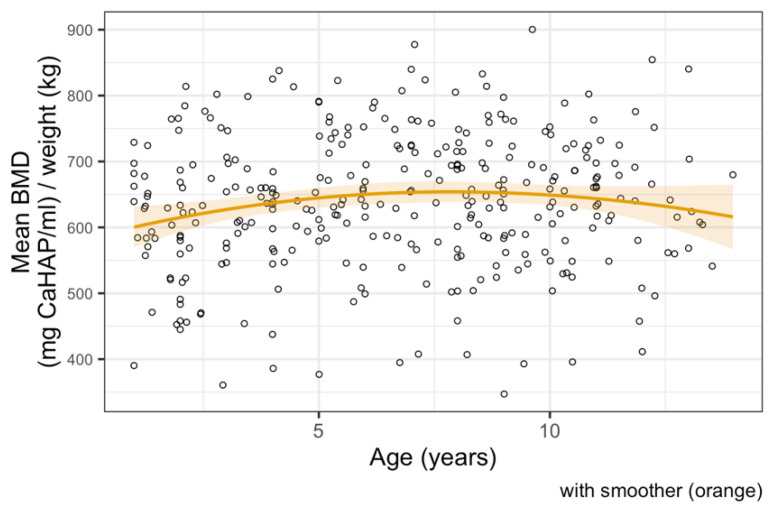
Linear regression model between bone mineral density and age, showing no correlation.

**Figure 5:**
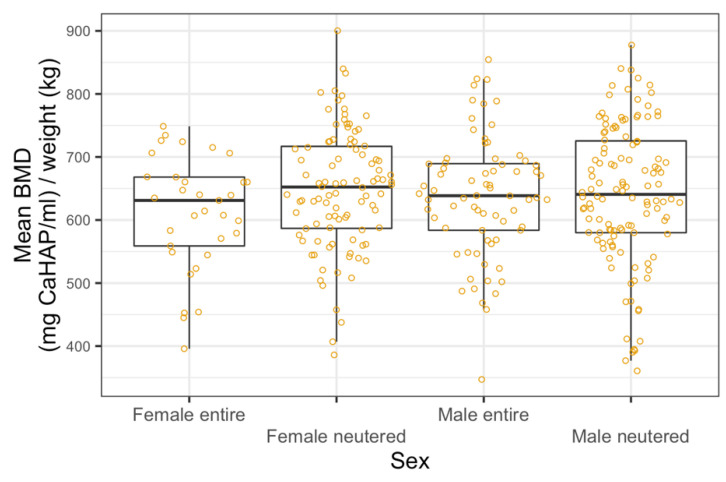
Linear regression model between bone mineral density and sex categories, showing no correlation.

The median BMD of all dogs in our study was 659 mg CaHAP/mL. The final linear regression model of mean BMD showed a negative relationship with weight; mean BMD decreased by 1.64 units (95% CI 0.67–2.61, *p* < 0.001) per kg increase in weight (Figure 3). Age and sex were not significant predictors and the relationship appeared to be approximately linear over the available data (Figure 4 and Figure 5). 

## 4. Discussion

In this study, we report a novel qCT approach to assess BMD using lamellar bone of the canine calvarium. 

Our hypotheses that osteodensitometric qCT of the canine head would be fast to perform in sedated or anaesthetized dogs; that BMD results are easy and quick to calculate; and that lower weight would be negatively associated with BMD were confirmed. Our hypotheses that increased age, female and neutered dogs would be negatively associated with BMD were not confirmed.

Although unreported in veterinary literature, the validity of measuring the skull bones for assessment of bone density has been well explored in the human field [25,26,27,28]. Bone mineral density of the skull bones is well correlated to the rest of the skeleton [28]. The measurement of BMD from the vertebral column, hips and wrists in people carry a number of technical uncertainties that make skull bone density measurements more appealing [29,30,31,32]. The qCT imaging technique featured in this paper was easy to perform and results obtained were in keeping with previous cadaveric studies [33]. 

Canine body weight was negatively correlated with BMD in this study. The relationship between weight and BMD is heavily underpinned by the complex interaction between adipose and osteoblast cells [34]. A recent study in humans has documented that abdominal fat and bone marrow adipose tissue are associated with increased cortical porosity and a reduced rate of bone formation [35]. As body condition score of dogs was not performed conclusions are based on absolute patient weight and associations between BMD and lean body weight cannot be assessed.

In contrast to findings of this study, BMD of the head decreases with age in humans [28]. Bone is a dynamic structure that is continually remodeling. With increasing age there is a distinct shift toward greater bone resorption and less bone synthesis. Many extrinsic factors contribute to BMD in aging humans and animals including diet, exercise and drugs [30,31]. In our study, dogs receiving medication known to affect bone health were excluded, but our study design did not allow us to assess diet or accurately deduce exercise level. 

In humans, the difference in BMD between men and women is stark [36]. Estrogen plays a vital role in female bone health. Decreased estrogen hastens bone loss and significantly decreases BMD in women [37]. Results of this study failed to show a similar relationship when comparing sex. Furthermore, neuter status did not contribute toward a difference in BMD between male and female dogs. This was an unexpected finding as estrogen and the effects of gonadectomy on bone density have been previously documented in dogs [38]. The exact date of gonadectomy was unknown in the dogs within this population. The effect of gonadal steroid deficiency on bone mineral density is not instantaneous. This could account for a proportion of neutered dogs retaining normal BMD and lack of significant difference.

The technique featured in this study measured lamellar BMD using qCT. In human patients both DEXA and qCT focus on assessment of trabecular bone. The trabecular bone is the metabolically most active compartment of bone and sensitive to systemic disease [39]. That being said, in humans, trabecular bone lacks uniformity [39] and in turn incurs BMD measurement uncertainty. Given that the calvarium consists of mainly homogenous lamellar bone similar to cortical bone [25], that cortical bone is affected by age related changes [39] and that a strong correlation exists between skull and whole body BMD in humans [28], the use of lamellar bone in this study was justified. However, future studies are needed to compare BMD obtained from canine lamellar and trabecular bone.

The median BMD in our study population was similar to cortical BMD reported in previous studies [33,40]. The calvarium is formed from intramembranous ossification [41]. Intramembranous ossification creates a tight, interwoven matrix of osteoblasts and colloid which is denser than bones arising from endochondral ossification which are percolated with chondroblasts and fibrous tissue. In humans the porosity of cortical bone is predictive of fractures [42]. Cortical bone is only affected by most severe metabolic disease [42] and therefore makes it less likely that subclinical disorders of dogs enrolled into the study affected BMD results.

In recent years, research in human medicine has moved away from DEXA scans and become focused on measuring BMD using qCT [43,44]. This has been promoted in order to reduce additional radiation exposure. Thus, CT scans have become an opportunity to obtain a global assessment of BMD. It is accepted that CT supersedes DEXA scans in veterinary patients when extreme variety of patient sizes and soft tissue dimensions render DEXA values inaccurate [45]. Veterinary publications that address qCT BMD are limited by small case numbers, use of cadavers, or use of a research population [14,40,46,47,48,49]. Our study contains a large population of clinical dogs and highlights that BMD can be assessed almost seamlessly in dogs undergoing CT for other clinical indications.

Computed tomography has become a gold standard for many conditions and therefore is now frequently performed in dogs, in particular for the head [50]. CT units used in veterinary practice are designed for human patients and have integrated osteodensitometry applications. Our study demonstrates that these integrated applications can be used in dogs undergoing head CT under sedation or general anesthesia quickly and are easily adaptable for the canine calvarium. This opens many new opportunities for screening of bone health status and bone metabolism in dogs, associated prognostic criteria and monitoring of treatment regimes. The fact that 10.7% of studies had an insufficient image quality to measure BMD predominantly due to positioning errors emphasizes the importance of operator training and quality control regimes.

There were a number of limitations in this study. The gold standard for BMD measurement is bone ash analysis [6,51] which could not be integrated in this clinical study given client-owned dogs rarely undergo postmortem examination. Despite a large number of dogs involved in this study, the total number of dogs of each breed was generally small and therefore the authors were unable to conclude any reliable breed-specific BMD results. Without a breed-specific BMD assessment, the authors advise cautious interpretation of osteodensitometry values based on the empirical weight groups chosen for this study. Previous studies in humans and dogs have predominantly used trabecular bone of lumbar vertebrae for BMD analysis. It was not feasible within the context of this study to add lumbar measurement to the head qCT performed, and there was no consistently measurably trabeculated bone in the skull. Cortical and presumably lamellar bone is less metabolically active than trabecular bone [39]. This may account for the negligible effect of age and sex on BMD in this study. Furthermore, there was a large range of dog breeds involved in this study, all with varied diets and exercise regimes. These factors could significantly alter cortical bone density and limits our ability to draw on cortical and trabecular bone correlation in humans, where these factors were better controlled. Inter-operator variability of BMD calculation was not measured in this study. Although dogs participating in this study showed no obvious clinical signs of bone disease, dogs were not systematically screened for diseases affecting bone metabolism. It is therefore possible that dogs suffering from diseases impacting on BMD were included in the study and the authors recommend cautious interpretation of these preliminary results. 

## 5. Conclusions

In conclusion, this study revealed that skull-based qCT in sedated or anaesthetized dogs is a practical diagnostic test that can be added with minimal additional time to head CT studies. Using the semiautomatic tracing software integrated in CT units for the canine lamellar calvaria bone, BMD can be measured easily and quickly. The calvarium of bone healthy dogs has a median BMD of 659 mg CaHAP/mL, and BMD is negatively correlated with weight, but there is no correlation with age, sex or neutered status.

## Data Availability

All relevant data for this study has been presented in this article. Raw data is available on request from the corresponding author.
